# Development of a novel peptide to prevent entry of SARS-CoV-2 into lung and olfactory bulb cells of hACE2 expressing mice

**DOI:** 10.1186/s13041-022-00956-1

**Published:** 2022-08-09

**Authors:** Ping Su, Dongxu Zhai, Albert H. C. Wong, Fang Liu

**Affiliations:** 1 Campbell Family Mental Health Research Institute, Centre for Addiction and Mental Health, 250 College Street, Toronto, ON M5T 1R8 Canada; 2 Department of Psychiatry, University of Toronto, Toronto, ON M5T 1R8 Canada; 3 Department of Physiology, University of Toronto, Toronto, ON M5S 1A8 Canada; 4 Institute of Medical Science, University of Toronto, Toronto, ON M5S 1A8 Canada; 5 Department of Pharmacology, Faculty of Medicine, University of Toronto, Toronto, ON M5S 1A8 Canada

**Keywords:** Angiotensin-converting enzyme 2 (ACE2), COVID-19, Interfering peptide, SARS-CoV-2, Viral entry

## Abstract

Severe acute respiratory syndrome coronavirus 2 (SARS-CoV-2) is a novel coronavirus that has caused a global pandemic Coronavirus Disease 2019 (COVID-19). Currently, there are no effective treatments specifically for COVID-19 infection. The initial step in SARS-CoV-2 infection is attachment to the angiotensin-converting enzyme 2 (ACE2) on the cell surface. We have developed a protein peptide that effectively disrupts the binding between the SARS-CoV-2 spike protein and ACE2. When delivered by nasal spray, our peptide prevents SARS-CoV-2 spike protein from entering lung and olfactory bulb cells of mice expressing human ACE2. Our peptide represents a potential novel treatment and prophylaxis against COVID-19.

**Supplementary Information:**

The online version contains supplementary material available at 10.1186/s13041-022-00956-1.

## Introduction

SARS-CoV-2 is the cause of a global pandemic (COVID-19), which has resulted in many deaths and unprecedented restrictions on travel, commerce and social interaction [1]. Currently, there are no effective treatments specifically for COVID-19 infection. SARS-CoV-2 is an RNA virus that attacks mucosal epithelial cells in the respiratory tract through binding the angiotensin-converting enzyme 2 (ACE2) and then fusing with the cell membrane to gain entry [2–4]. There are three steps in this process. (1) Binding of spike proteins (S) embedded in the outer SARS-CoV-2 lipid membrane to cellular ACE2 [2, 4]. (2) The proprotein convertase furrin cleaves the S protein at the S1/S2 site, and transmembrane protease serine 2 (TMPRSS2), an S1 family serine protease on the cell surface, cleaves the S protein at the S2’ site [2, 5–7]. (3) The remaining spike fragments include two domains in the S2 subunit (heptad repeat 1 and 2: aa 910–988 and aa 1162–1206) that form a six-helix bundle (6-HB) fusion core [8, 9]. This transient protein structure brings the viral and cellular membranes together, allowing them to fuse and enabling viral RNA to enter cells [8, 10]. Viral RNA entry is the first step in the cycle of infection [11].

Each of the three steps require different proteins to interact with each other at a specific site that has already been identified [2, 9, 12–15]. These types of protein–protein interactions can be blocked by small polypeptides that mimic critical portions of the interacting site as previously described by our lab and the other labs in the world [16–28]. Because the interacting sites between ACE2, TMPRSS2, SARS-CoV-2-S, and within the fusion core are known [2, 9, 12–15], we sought to develop peptides mimicking these sites to block these interactions, with the goal of preventing viral entry. Using this strategy, we have developed a protein peptide that effectively disrupts the binding between the SARS-CoV-2 spike protein and ACE2. When delivered by nasal instillation, our peptide prevents SARS-CoV-2 spike protein from entering lung and olfactory bulb cells of mice expressing human ACE2. Our peptide represents a potential novel treatment and prophylaxis against COVID-19.

## Results

### TAT-fused peptides block S-ACE2 interaction or cleavage of S protein

**Fig. 1:**
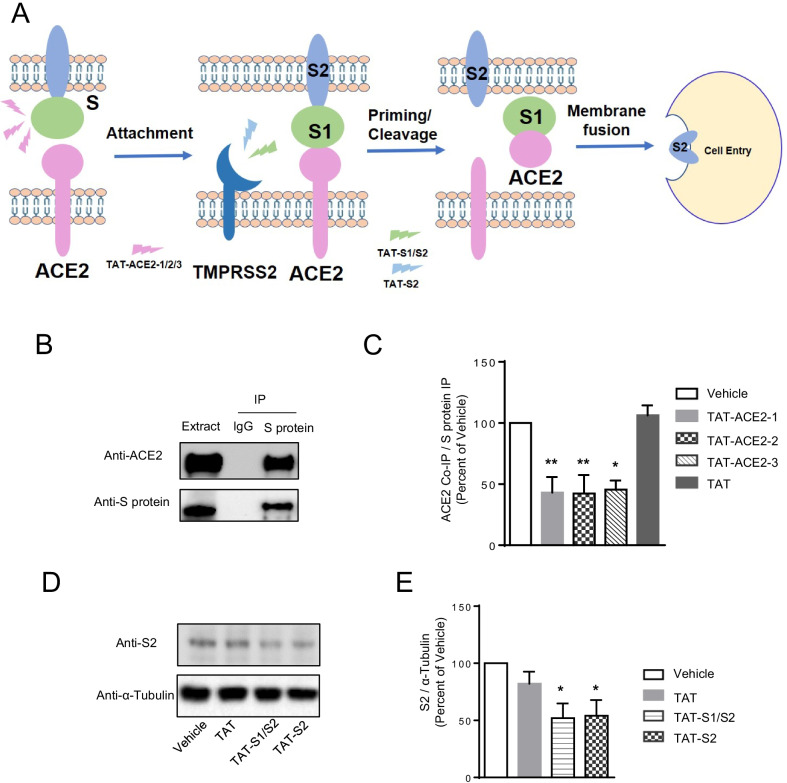
Peptides are able to block the interaction between ACE2 and SARS-CoV-2 spike protein, or inhibit the cleavage of spike protein by protease. **A** Schematic diagram of design strategy for peptides to block SARS-CoV-2 virus from entering cells. **B** Representative blot image showing that ACE2 can be co-immunoprecipitated by anti-Spike protein (S protein) antibody, but not IgG, in HEK293T cells transfected with S protein and hACE2. **C** Densitometric analysis of the levels of ACE2 co-immunoprecipitated by anti-S protein antibody in HEK293T cells transfected with S protein and ACE2 shows that TAT-ACE2-1, TAT-ACE2-2 and TAT-ACE2-3 peptides, but not TAT alone, are able to decrease the level of S-ACE2 complex. The level of co-immunoprecipitated ACE2 (ACE2 Co-IP) was normalized to the level of precipitated S protein (S protein IP). Data are shown as mean ± SEM, and presented as the percentage of the control (vehicle) sample. n = 3 independent experiments for each group, one-way *ANOVA* test followed by Dunnett’s post hoc test. *p < 0.05, **p < 0.01 as compared to control (vehicle) samples. **D** Western blot shows that TAT-S1/S2 or TAT-S2 peptides are able to inhibit S protein cleavage by TMPRSS2 in HEK293T cells transfected with S protein, ACE2 and TMPRSS2. α-Tubulin was used as a loading control. **E** Densitometric analysis of the levels of S2 in HEK293T cells transfected with S protein, ACE2 and TMPRSS2, and treated with either vehicle (control), TAT alone, TAT-S1/S2 or TAT-S2 peptides. The level of S2 was normalized to the level of α-Tubulin. Data are shown as mean ± SEM, and presented as the percentage of the control (vehicle) sample. n = 3 independent experiments for each group, one-way *ANOVA* test followed by Dunnett’s post hoc test. *p < 0.05 as compared to control (vehicle) samples

We synthesized two groups of interfering peptides that are analogues of the interacting sites that allow the binding of the spike protein (S protein) to ACE2 and the TMPRSS2-mediated cleavage of S protein based on the amino acid sequence of the interacting domains [2–4, 8–15]. As shown in Fig. 1A, three Group 1 peptides were designed to block binding of ACE2 to S protein based on the ACE2 sequence: ACE2-1:I21-S43, ACE2-2:A71-N90, and ACE2-3:Q325-R357 [12]. Group 2 peptides were designed to block TMPRSS2 cleavage/priming, and include two peptides based on the S protein sequence: S1/S2: T676-A688 and S2: K811-I818 [2]. To promote the cellular uptake of these peptides, they were fused to the cell-membrane transduction domain of the human immunodeficiency virus-type 1 (TAT), as previously described [16, 17, 22, 27].

We hypothesized that these interfering peptides could block the attachment or priming of SARS-CoV-2-S proteins, demonstrating their potential as therapeutic or prophylactic agents specific to this new coronavirus. Our first step was to confirm whether S protein and ACE2 form a protein complex using co-immunoprecipitation assays with HEK-293T cells expressing S protein and human ACE2 (hACE2). As predicted, antibodies against S protein can co-immunoprecipitate hACE2, suggesting the existence of hACE2-S protein complex in our in vitro cellular model system (Fig. 1B). We then tested whether group 1 peptides could block the hACE2-S protein interaction under the same experimental conditions. As shown in Fig. 1C, pre-treatment with TAT-ACE2-1, TAT-ACE2-2, or TAT-ACE2-3 (10 µM, 24 h), but not vehicle or TAT alone, significantly reduced the immunoprecipitation of hACE2 with antibodies against S protein, compared to the vehicle or TAT alone negative controls. ACE2 levels showed no significant differences in any of the treatment groups (Additional file 1: Fig. S1A, B). This experiment demonstrated that these three peptides are effective in blocking the S protein-hACE2 interaction.

We next tested whether TAT-S1/S2 and TAT-S2 are able to block TMPRSS2 cleavage of S protein in HEK293T cells transfected with S protein, hACE2 and TMPRSS2 plasmids [2]. Cells were treated with TAT-S1/S2_,_ TAT-S2, TAT alone peptides or vehicle, and TMPRSS2-mediated S protein cleavage into S1 and S2 subunits was detected with Western blots using antibody against the S2 terminus of S protein. As shown in Fig. 1D, E, the expression of S2 subunit is significantly decreased in cells treated with TAT-S1/S2 or TAT-S2 peptides compared to cells treated with vehicle or TAT alone, and the ratio of S2/S was significantly decreased with treatment of TAT-S1/S2 or TAT-S2 compared to the control treatments with either vehicle or TAT (Additional file 1: Fig. S1C, D), while the expression levels of the full-length S protein and the S2 fragment remain unchanged across the treatment groups (Additional file 1: Fig. S1C, E), suggesting that both TAT-S1/S2 and TAT-S2 treatment blocked TMPRSS2-mediated S protein cleavage.

### TAT-fused peptides inhibit SARS-CoV-2 S protein-mediated cell–cell fusion

**Fig. 2:**
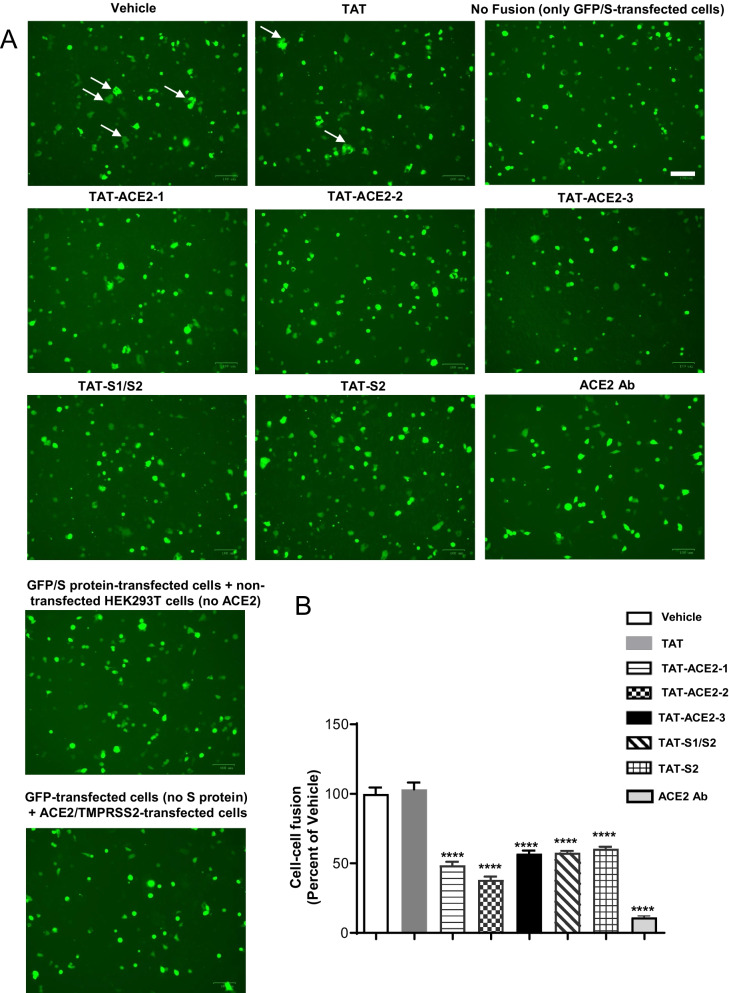
TAT-peptides inhibit SARS-CoV-2 S protein-mediated cell–cell fusion in HEK293T cells. **A** Sample microscopic images of SARS-CoV-2 S protein-mediated cell–cell fusion in HEK293T cells treated with either vehicle (Control), ACE2 antibody (2 µg/ml, 1 h), TAT alone, TAT-ACE2-1, TAT-ACE2-2, TAT-ACE2-3, TAT-S1/S2, or TAT-S2 peptides at 10 µM (scale bar: 100 µm). White arrows indicate the fused cells. **B** TAT-ACE2-1, TAT-ACE2-2, TAT-ACE2-3, TAT-S1/S2, TAT-S2, or ACE2 antibody each inhibit SARS-CoV-2 S protein-mediated cell–cell fusion. The number of fused cells was expressed as a fraction of total cell number in the same image. Data are shown as mean ± SEM, and presented as the percentage of the control (vehicle) samples. n = 10 – 21 images for each group, one-way *ANOVA* test followed by Dunnett’s post hoc test. ****p < 0.0001 as compared to control (vehicle) samples

After binding of S protein to hACE2 and TMPRSS2-mediated cleavage of S protein, the next step in SARS-CoV-2 infection is formation of the fusion core that enables viral RNA to enter cells [8–10]. Thus, we examined the effect of our peptides on cell–cell fusion assays in HEK-293T cells co-transfected with SARS-CoV-2 S protein and GFP (effector cells), and HEK-293T cells expressing hACE2 and TMPRSS2 (target cells) [9, 29]. Effector cells were detached 40 h post transfection and overlaid on an 80% confluent monolayer of target cells at an approximately 1:3 ratio [29]. Fused cells can be directly observed with fluorescence microscopy. As shown in Fig. 2A, B, all five peptides inhibited effector and target cell fusion (Fig. 2A, B). Effector cells alone were used as a negative control (no fusion) [9], and cells treated with vehicle were used as the positive control. Furthermore, we also tested cell–cell fusion in two more control conditions: (1) GFP/S-transfected cells + non-transfected HEK293T cells (no ACE2); (2) GFP-transfected cells (no S protein) + ACE2/TMPRSS2-transfected cells. As shown in Fig. 2A, there were no fusions in these two conditions, showing that cell fusion is mediated by the interaction between ACE2 and S protein. Additionally, we added an ACE2 antibody treatment group (2 μg/ml, 1 h), and cell–cell fusion was also blocked by this antibody (Fig. 2A, B). These data confirm that SARS-CoV-2 S protein-mediated cell–cell fusion was inhibited by the TAT-fused peptides.

### TAT-fused peptides block cell entry of pseudovirus containing SARS-CoV-2 S protein

**Fig. 3:**
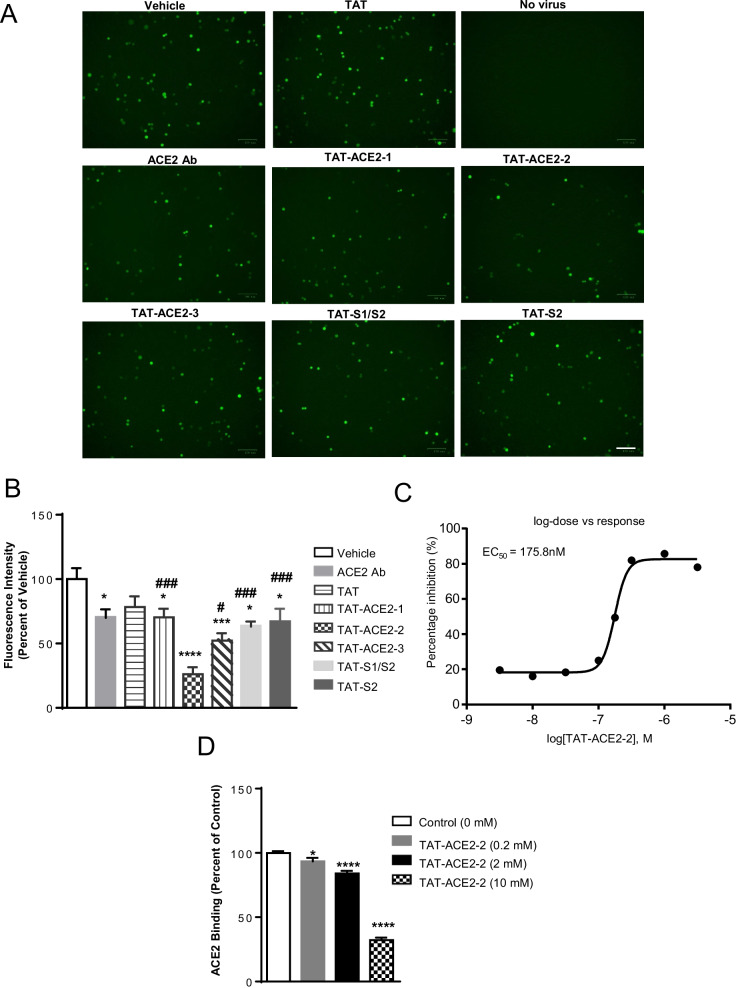
TAT-peptides inhibit SARS-CoV-2 S protein entry into HEK293T cells. **A** Representative fluorescent microscopy images are shown. HEK293T cells transiently transfected with hACE2 and TMPRSS2 were pre-incubated with vehicle, ACE2 antibody, TAT alone or related TAT-peptides (10 µM), and inoculated with pseudotyped VSV expressing SARS-CoV-2 S protein. Pseudotyped VSV contains a GFP reporter gene. ACE2 antibody was used as a positive control. Scale bar: 100 µm. **B** TAT-ACE2-1, TAT-ACE2-2, TAT-ACE2-3, TAT-S1/S2, or TAT-S2 inhibit the SARS-CoV-2 S protein entry into HEK293T cells. The fluorescent intensity of GFP in live cells was quantified using a Synergy H4 plate luminometer. Data are shown as mean ± SEM, and presented as the percentage of the control (vehicle) samples. n = 8 – 14 samples for each group, one-way *ANOVA* test followed by Dunnett’s post hoc test. *p < 0.05, ***p < 0.001, ****p < 0.0001 as compared to control (vehicle) samples. #p < 0.05, ###p < 0.001 as compared to TAT-ACE2-2 samples. **C** Analysis of EC50 of TAT-ACE2-2 peptide for inhibiting VSV-ΔG-GFP-SARS-CoV-2 pseudovirus entry into HEK 293 T cells transfected with hACE2 and TMPRSS2. Eight dilutions ranging from 3 nM to 3 µM were assessed. **D** TAT-ACE2-2 inhibits the binding of purified S protein and ACE2. The RBD domain of SARS-CoV-2 S protein was coated onto a 96-well plate, and the same concentration of purified ACE2 together with various concentrations of TAT-ACE2-2 were simultaneously added into each well. ACE2 binding to S protein was detected using anti-ACE2 antibody and HRP-conjugated secondary antibody; TMB one-step substrate reagent was added to visualize HRP. Data are shown as mean ± SEM, and presented as the percentage of the control samples (no TAT-ACE2-2 added, 0 mM). n = 3 samples for each group; one-way *ANOVA* test followed by Dunnett’s post hoc test. *p < 0.05, ****p < 0.0001 as compared to control (0 mM) samples

To extend these promising results, we moved to the more realistic model system of replication-defective VSV particles expressing SARS-CoV-2 S protein with a GFP reporter and HEK 293T cells expressing hACE2 and TMPRSS2 [2]. The VSV pseudovirus has the SARS-CoV-2 spike protein on its surface, but is restricted to a single round of replication for safety [30]. We chose this model to test whether our peptides can block viral entry, the critical initial event in SARS-CoV-2 infection. HEK293T cells expressing hACE2 and TMPRSS2 were treated with 5 μM of each interfering peptide 1 h prior to pseudoviral infection and 5 μM again at the same time as pseudoviral infection at an MOI (multiplicity of infection) of 2. Viral entry into target cells was tested by measuring the intensity of GFP fluorescence in live cells using a Synergy H4 plate luminometer (Biotek). hACE2 antibody was used as the positive control [2]. At 24 h post-infection, all of our peptides inhibited viral entry into target cells, but TAT-ACE2-2 was the most effective (Fig. 3A, B). We further examined the ability of TAT-ACE2-2 to block viral entry at various concentrations. As shown in Fig. 3C, viral entry was decreased by TAT-ACE2-2 in a concentration-dependent manner, with an estimated EC50 of 175.8 nM. In addition, using the RayBio® COVID-19 Spike-ACE2 binding assay, we further characterized the binding of S-hACE2 in the presence of various concentrations of TAT-ACE2-2. The RayBio® COVID-19 Spike-ACE2 binding assay consists of a 96-well plate coated with recombinant S-protein receptor binding domain (S-RBD). TAT-ACE2-2 peptide was then added in the presence of purified recombinant hACE2 protein. As shown in Fig. 3D, S-ACE2 binding was inhibited by TAT-ACE2-2 in a concentration-dependent manner.

### TAT-ACE2-2 prevents entry of pseudovirus containing SARS-CoV-2 S protein into lung and olfactory bulb cells in hACE2 expressing mice

**Fig. 4:**
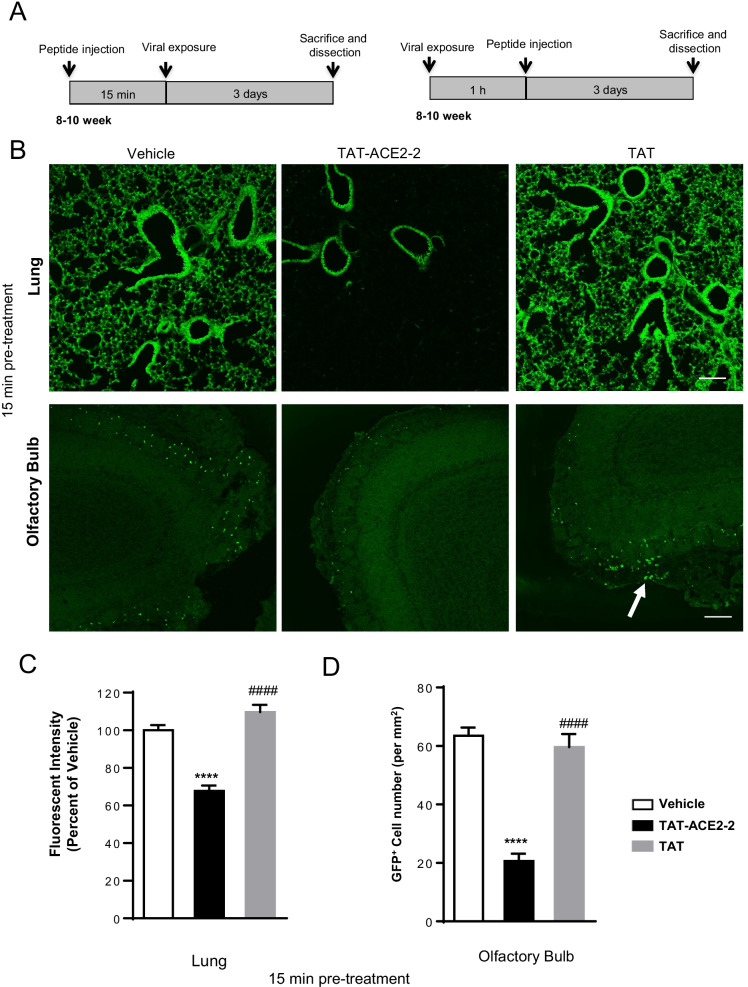

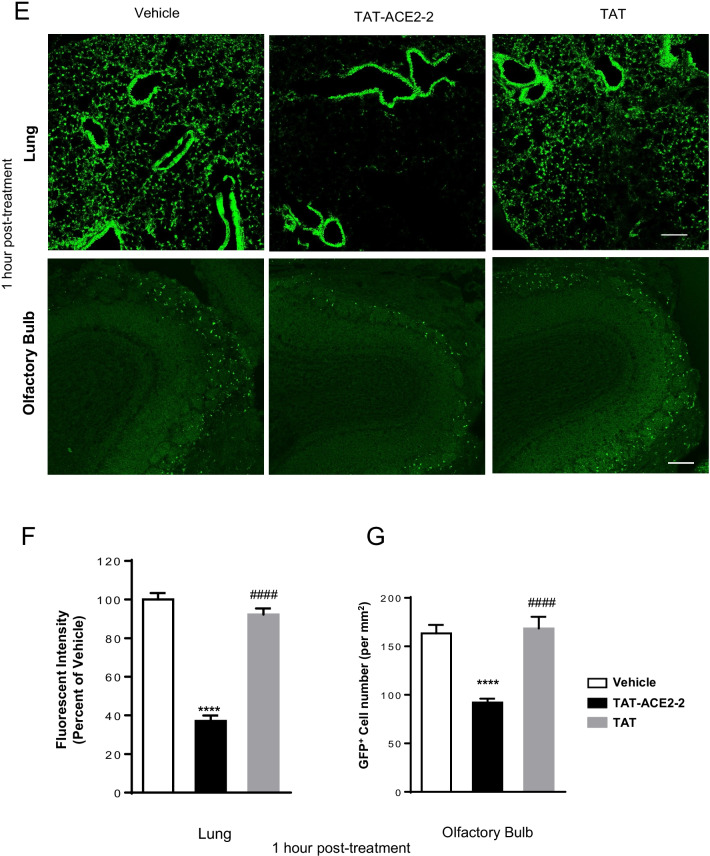
Pre- and post-treatment effects of TAT-ACE2-2 peptide on lung and olfactory bulb tissue in hACE2 transgenic mice infected with VSV-ΔG-GFP-SARS-CoV-2 pseudovirus. **A** Schematic diagram of peptide injection and viral exposure in hACE2 mice. **B** Representative fluorescent microscopic images of lung tissue and olfactory bulb treated with TAT-ACE2-2 peptide, vehicle and TAT alone peptide intranasally 15 min before viral exposure. The white arrow indicates the GFP^+^ cells (pseudovirus-infected cells). Scale bar: 150 µm. **C** Bar graph showing significantly less fluorescence in the lung tissue treated with TAT-ACE2-2 peptide 15 min before viral exposure as compared to vehicle and TAT alone groups. Data are shown as mean ± SEM, and presented as the percentage of the vehicle group. n = 34 – 48 images from 4–5 mice for each group, one-way *ANOVA* test followed by Tukey’s post hoc test. ****p < 0.0001 as compared to vehicle group, ####p < 0.0001 as compared to TAT-ACE2-2 group. **D** Bar graph showing significantly lower total counts of GFP^+^ cells in the OB in the TAT-ACE2-2 peptide-treated group compared to vehicle and TAT alone groups when treated 15 min before viral exposure. Data are shown as mean ± SEM. n = 23 – 49 images from 4–5 mice for each group, one-way *ANOVA* test followed by Tukey’s post hoc test. ****p < 0.0001 as compared to vehicle group, ####p < 0.0001 as compared to TAT-ACE2-2 group. **E** Representative fluorescent microscopic images of lung tissue and olfactory bulb treated with TAT-ACE2-2 peptide, vehicle and TAT alone peptide intranasally 1 h after viral exposure. Scale bar: 150 µm. **F** Bar graph showing that TAT-ACE2-2 treatment 1 h after viral exposure significantly decreases fluorescence in lung tissue as compared to vehicle or TAT alone groups. Data are shown as mean ± SEM, and presented as the percentage of the vehicle group. n = 52 – 65 images from 4–5 mice for each group, one-way *ANOVA* test followed by Tukey’s post hoc test. ****p < 0.0001 as compared to vehicle group, ####p < 0.0001 as compared to TAT-ACE2-2 group. **G** Bar graph showing that treatment with TAT-ACE2-2 1 h after viral exposure significantly decreases the number of GFP^+^ cells in the OB tissue as compared to vehicle or TAT alone groups. Data are shown as mean ± SEM. n = 20—26 images from 4–5 mice for each group, one-way *ANOVA* test followed by Tukey’s post hoc test. ****p < 0.0001 as compared to vehicle group, ####p < 0.0001 as compared to TAT-ACE2-2 group

To translate these in vitro results to in vivo, we tested whether TAT-ACE2-2 blocked VSV pseudovirus from entering cells in transgenic mice expressing hACE2 [31]. TAT alone was used as a negative control. Pseudotyped VSV-∆G-GFP-SARS-CoV-2 S virus was produced and concentrated to 1 × 10^9^ IU. As illustrated in Fig. 4A, the interfering peptide was delivered intranasally, 15 min prior to or 1 h after intranasal challenge with pseudovirus [32]. Viral entry was assessed 3 days after viral exposure by measuring GFP fluorescent intensity [32]. We first confirmed the presence of the TAT-ACE2-2 peptide in mouse lung slices, three days after intranasal administration (Additional file 1: Fig. S2). We then measured GFP intensity in mouse lung tissue three days after viral exposure. As shown in Fig. 4B (top panel)-4C, GFP signal was significantly decreased in mice treated with TAT-ACE2-2 15 min prior to viral exposure, confirming that disruption of S-ACE2 coupling also inhibits viral entry *in* *vivo*.

Altered olfactory function is a common symptom of COVID-19. Thus, we examined whether TAT-ACE2-2 blocks viral entry into cells of the olfactory bulb. We first investigated the cellular localization of the pseudovirus in the olfactory bulb. We found that pseudotyped VSV-∆G-GFP-SARS-CoV-2 S virus co-localized with ACE2 (Additional file 1: Fig. S3) and calbindin (CB) (Additional file 1: Fig. S4A, B) but not tyrosine hydroxylase (TH) (Additional file 1: Fig. S5). The colocalization of pseudovirus with CB suggested the presence of pseudovirus in CB-positive periglomerular cells in the olfactory bulb [33]. We also confirmed the presence of TAT-ACE2-2 in the olfactory bulb (Additional file 1: Fig. S6). We then treated mice with TAT-ACE2-2 peptide and counted GFP-positive cells in the olfactory bulb region three days after intranasal exposure to pseudovirus. As shown in Fig. 4B (bottom panel) and 4D, there were significantly fewer GFP-positive olfactory bulb cells in mice treated with TAT-ACE2-2 15 min prior to viral exposure*. *Similar effects were also observed in both lung and olfactory bulb region of mice treated with TAT-ACE2-2 1 h after viral exposure (Fig. 4E–G). Taken together, our data suggest that intranasal delivery of TAT-ACE2-2 peptide is capable of inhibiting viral entry into both lung and olfactory bulb cells.

## Discussion

Our findings are the first step towards developing a novel antiviral drug for treating and preventing COVID-19. Although the speed with which COVID-19 vaccines have been developed is remarkable (URL: https://www.who.int/publications/m/item/draft-landscape-of-covid-19-candidate-vaccines), they are still not available for widespread use [34]. The efficacy of these vaccines, especially with new viral strains, remains to be seen, and some patients will succumb to COVID-19 even with vaccination (URL: https://www.who.int/publications/m/item/draft-landscape-of-covid-19-candidate-vaccines) [35]. The coronavirus family has caused a series of recent epidemics: SARS, MERS and now COVID-19 [36]. Therefore, it is likely that new coronaviruses will emerge and cause disease in humans, making it vital to pursue treatments such as ours in combination with vaccines. Our proposed peptide therapeutic represents a new anti-viral strategy that could be the start of a novel family of treatments for coronaviruses that are likely to cause other epidemics in the future.

Peptide-based antivirals have shown promising efficacy against other respiratory viruses [37, 38] and this current project extends this concept with our particular expertise in peptide design, to specifically target COVID-19. We have previously delivered peptide therapeutics intranasally [27], and this or inhaled routes could specifically target the upper respiratory tract and lung that SARS-CoV-2 attacks, minimizing systemic exposure. This is important because oral delivery of peptides is limited by degradation in the stomach. Because these interfering peptides are so specific for their targets, small amounts can be used. Finally, therapeutic peptides could be applied in basically the form that was used in these experiments without the time-consuming additional refinement typical for conventional small molecule drugs.

A potential concern with our data is the TAT-peptide may become quickly degraded; hence, the signal we detected could represent an artifact from degraded fragments or single amino acids binding the fluorescent tag. There are several reasons to believe that this is not the case. Firstly, regardless of what epitope is being recognized by the fluorescently-tagged antibody, co-immunoprecipitation demonstrates the physical binding of the interacting protein. Thus, our data show that our interfering peptide reduces the protein–protein interaction in question compared to various negative control conditions. In addition, the methods used in this paper have been applied to a series of protein–protein interactions involving cell surface receptors and other interacting proteins over the past 20 years. While previous work was done in the context of neurotransmitter receptors, there is little reason to assume validity of the overall approach should differ for ACE2 [17–19, 22, 26, 28].

Our results suggest that TAT-ACE2-2 is effective in blocking viral entry when given shortly before or after viral exposure. The potential benefit of a therapeutic agent based on our peptide is especially relevant to two populations. Anyone infected with SARS-CoV-2 would benefit from treatment with our therapeutic peptides, as it could stop the spread of the virus throughout the lungs and the rest of the body. Our anti-SARS-CoV-2 peptide could thus reduce the severity and duration of COVID-19 infection. People with a high-risk exposure event such as health care workers and first responders, could also benefit from a prophylactic dose of peptide in addition to vaccination. Our peptide therapeutic could quickly move to clinical trials and has the potential to reduce morbidity and mortality from this global pandemic. Because the peptide design was based on the sequence of hACE2, it is likely to be effective against other coronaviruses, such as SARS (our peptides include the fragments of ACE2 that interacts with SARS spike protein) [39], that also bind ACE2 to infect the lung.

In summary, we have developed a protein peptide that effectively disrupts the binding between the SARS-CoV-2 spike protein and ACE2, and this peptide prevents SARS-CoV-2 spike protein from entering lung and olfactory bulb cells of mice expressing human ACE2 when delivered by nasal instillation. Our peptide represents a potential novel treatment and prophylaxis against COVID-19.

## Materials and methods

### DNA constructs and peptides

The plasmids encoding the SARS-CoV-2 spike protein (S protein) (pTwist-EF1 alpha-SARS-CoV-2-S-2xStrep was a gift from Nevan Krogan [40] (Addgene plasmid #141,382)), human ACE2 (hACE2 was a gift from Hyeryun Choe [41] (Addgene plasmid #1786; http://n2t.net/addgene:1786; RRID: Addgene_1786)), and TMPRSS2 (TMPRSS2 was a gift from Roger Reeves [42] (Addgene plasmid #53,887; http://n2t.net/addgene:53887; RRID: Addgene_53887)) were purchased from Addgene. After the bacteria were amplified, plasmids were purified using GenElute Plasmid Maxiprep Kit (Sigma-Aldrich). Peptides were synthesized by Biomatik, dissolved in DMSO to a stock solution of 10 mM for treating HEK293T cells and 50 mM for the in vitro blocking assay, or were dissolved in ethanol to a stock solution of 50 mM for for in vivo treatments in mice. For each intranasal treatment in mice, 12 µl of peptide stock solution was used.

### Cell culture and DNA transfection

HEK293T cells were maintained in Dulbecco’s Modified Eagle Medium (DMEM) (Gibco) supplemented with 10% fetal bovine serum (Gibco) at 37 °C. Cells that were grown to 70–80% confluency were transiently transfected with DNA constructs using X-treme gene 9 transfection reagent (Roche) following the manufacturer’s instructions, and. Cells were used for various experiments 24 or 48 h after transfection. Transfected cells were treated with either vehicle, TAT, or peptides 16 h after transfection, and 24 h after treatment, cells were treated again for 1 h before harvesting. For detection of the S-ACE2 interaction, cells were transfected with both S protein and hACE2. For testing of the cleavage of S protein, cells were transfected with S protein, hACE2, and TMPRSS2.

### Co-immunoprecipitation and Western blot

Co-immunoprecipitation and Western blot analyses were performed as previously described [18, 19, 22]. After treatment, cells were lysed with lysis buffer (50 mM Tris, 150 mM NaCl, 2 mM EDTA, 1% NP-40, 0.5% sodium deoxycholate, 1% Triton X-100, 0.1% SDS) with protease inhibitor cocktail (Sigma-Aldrich). After shaking at 4 °C for 1 h, the mixture was centrifuged at 10,000 g for 10 min at 4 °C, and the supernatant was collected as the total protein extract. Protein concentration was quantified by BCA protein assay (Pierce). For co-immunoprecipitation, 800 µg of cellular protein extract was incubated in the presence of primary antibody against S protein or control IgG (3 µg) as well as 25 µl of protein A/G plus agarose (Santa Cruz Biotechnology) for 12 h at 4 °C. Pellets were washed, boiled for 5 min in SDS sample buffer and subjected to SDS-PAGE. 5–50 µg of cellular protein extract was used as the control in each experiment. The blots were imaged using the ChemiDoc MP Gel Imaging System (Bio-Rad) and the densitometric analysis was conducted using ImageLab (Bio-Rad). Antibodies used for co-immunoprecipitation and Western blot include: anti-SARS-CoV-2 spike antibody (GeneTex, mouse, Cat No. GTX632604), anti-hACE2 (R&D Systems, goat, Cat No. AF933), anti-α-Tubulin (Sigma-Aldrich, mouse, Cat No. T8203), normal mouse IgG (Santa Cruz Biotechnology, Cat No. sc-2025), HRP-conjugated anti-goat secondary antibody (Thermo Fisher Scientific, Cat No. A15999), and HRP-conjugated anti-mouse secondary antibody (Cell Signaling Technology, Cat No. 7076).

### In vitro testing of S-ACE2 binding

To assay binding of purified ACE2 and S protein in vitro, we used the RayBio® COVID-19 Spike-ACE2 binding assay kit. Briefly, the RBD domain of SARS-CoV-2 S protein was coated onto a 96-well plate, and the same concentration of purified ACE2 was added together with different concentrations of TAT-ACE2-2 (0, 0.2, 2, and 10 mM) simultaneously to each well. ACE2 binding to S protein was detected using anti-ACE2 antibodies and HRP-conjugated secondary antibodies, and visualized with TMB one-step substrate reagent that reacts with HRP. The reaction was stopped with stop solution, and absorbance at 450 nm was measured with the Synergy H4 plate luminometer (Biotek).

### Cell–cell fusion assay

Cell–cell fusion assays were conducted as previously described [9, 29]. Briefly, HEK293T cells transiently transfected with hACE2 and TMPRSS2 were used as target cells, and HEK293T cells transiently transfected with SARS-CoV-2 S protein and GFP were used as effector cells. 40 h after transfection, target cells were treated with vehicle, TAT or peptide (10 µM) 15 min before adding the effector cells. The effector cells were detached from their culture dishes with 0.25% trypsin and overlaid onto a target cell monolayer at an effector:target cells ratio of 1:3. After 2 h incubation, 10–21 images were randomly taken to count the number of fused *vs.* unfused cells using fluorescence microscopy (Bio-Rad). The cells number were counted using ImageJ (NIH). Effector cells incubated in DMEM supplemented with 10% FBS without target cells were used as a negative control. All experiments were performed in triplicate. This assay was performed in a double-blind fashion.

### VSV-ΔG-SARS-CoV-2 pseudovirus packaging and concentration

The VSV-ΔG-GFP Plasmid Expression Vector was purchased from Kerafast INC (EH1004 and EH1019). The pTwist-EF1 alpha-SARS-CoV-2-S-2xStrep vector was purchased from Addgene (141,382). To generate high titer stocks of VSV-ΔG-GFP, HEK 293 T cells were transfected with VSV-G helper vector (EH1012, Kerafast INC) using X-treme Gene HP transfection reagent (Roche) for 24 h, then infected with Pseudotyped ΔG-GFP (G*ΔG-GFP) rVSV virus at an MOI of 0.1. The supernatants were collected after 48 h and clarified by centrifugation at 1000×*g* for 7.5 min and stored at − 80℃. HEK 293 T cells were plated on a T75 flask in DMEM + 10% FBS at 100,000 cells/cm^2^. After 6–8 h, 13 μg of pTwist-EF1alpha-SARS-CoV-2-S-2xStrep were transfected into these HEK 293 T cells using X-treme Gene HP transfection reagent according to the procedure recommended by the manufacturer. Cells were then infected with Pseudotyped ΔG-GFP (G*ΔG-GFP) rVSV virus at an MOI of 2. The transfected cells were incubated at 37℃ and 5% CO2 overnight. The supernatant containing pseudovirus was collected after 24 h and passed through a 0.45 μm filter and stored at -80℃ for later use or at 4℃ for immediate use [43].

To concentrate the VSV-ΔG-SARS-CoV-2 pseudovirus, Beckman Ultra-Clear centrifuge tubes (Cat # 344,058) were first sterilized for 15 min with UV light in a biological safety cabinet. Supernatant was added to these tubes and centrifuged at 100,000 rpm for 90 min at 4 °C in a Beckman SW32Ti rotor. Following centrifugation, the supernatant was carefully decanted and discarded, leaving the final 1 ml, which was kept and pooled with other tubes for the final round of centrifugation with the same parameters. Pellets were then resuspended and kept overnight at 4℃ or stored at − 80℃.

### Assays of VSV-ΔG-SARS-CoV-2 pseudoviral entry

White opaque 96-well plates (Sarstedt) were coated with 0.2 mg/ml poly-D-lysine (Sigma-Aldrich, P0899) overnight at 37℃. HEK293T cells were plated into the wells at the density of 2 × 10^4^ cells per well in 200 µl DMEM supplemented with 10% FBS. The cells were transiently transfected with hACE2 and TMPRSS2. 24 h after transfection, cells were treated with vehicle, TAT, or peptides at 5 µM 1 h before adding pseudovirus. After 1 h, the cells were treated for the second time with vehicle, TAT, or peptides at 5 µM, and the VSV-ΔG-SARS-CoV-2 pseudovirus was added immediately. 24 h later, fluorescent intensity in each well was quantified using the Synergy H4 plate luminometer (Biotek) and photographed through the microscope (Bio-Rad).

### Animal experiments

All animal experimental procedures were conducted in accordance with the protocol (#849) approved by the Animal Care Committee in Centre for Addiction and Mental Health.

Eight to ten week-old B6.Cg-Tg (K18-ACE2)2Prlmn/J mice were purchased from Jackson laboratories (Stock #034860). Mice were housed in ventilated cages in a controlled environment that included standard enrichment. Animals were closely monitored for health and well-being daily by the investigator and supervised by a certified veterinarian in accordance with standard animal care guidelines by CACC (Canadian Council on Animal Care). Animals were acclimatized to the environment for at least 7 days before experiments. Pseudoviral work, including intranasal inoculation, were performed in a biosafety risk level 2 + laboratory. Mice were anesthetized with isoflurane using a vaporizer (Benson Medical Industries INC, 5% at induction). TAT-ACE2-2 peptide, vehicle or TAT peptide were delivered intranasally 15 min before or 1 h after intranasal pseudovirus inoculation (1 × 10^7^ VSV-ΔG-GFP-SARS-CoV-2 pseudovirus in10 µl). Pseudovirus was delivered with a micropipette with gel-loading tips in both nostrils. After 72 h, animals were sacrificed and perfused with PBS followed by 4% paraformaldehyde (PFA) solution. Lung and olfactory bulb were dissected and fixed in 4% PFA for 48 h followed by 20% and 30% sucrose for another 48 h before cryostat sectioning. Tissues were cut into 25 µm slices and examined under confocal microscopy (FV1200, Olympus INC). The fluorescent intensity in lung tissue and GFP-positive cell numbers in olfactory bulb tissue were analyzed using ImageJ (NIH).

### Immunohistochemistry

Frozen coronal sections of olfactory bulb were initially permeabilized with PBS and 0.3% Triton X-100 (Sigma-Aldrich) for 30 min, and incubated with blocking solution (0.3% Triton X-100 and 3% BSA (Sigma-Aldrich) in PBS) for 2 h at room temperature to reduce nonspecific background. The sections were then incubated with primary antibodies and diluted in PBS containing 0.3% Triton and 1% BSA overnight at 4℃. After this, the sections were washed with PBS for 3 times, and incubated with secondary antibodies for 2 h at room temperature. Finally, the sections were mounted using Prolong Gold Antifade Mountant (Thermo Fisher Scientific). The primary antibodies used were: anti-calbindin (1:30, rabbit, Cat No. ab108404, Abcam), and anti-hACE2 (1:150, goat, Cat No. AF933, R&D Systems). Fluorescent secondary antibodies conjugated to either Alexa 594 (1:200, Invitrogen) were used to detect the primary antibodies. DAPI (Invitrogen) was used to stain the nuclei.

### Statistical analyses

Data were analyzed by one-way ANOVA test followed by Dunnett’s or Tukey’s post hoc test using GraphPad Prism (GraphPad Software). For EC50 analysis, data were analyzed by the nonlinear curve fit module using GraphPad Prism (GraphPad Software).

## Supplementary Information

**Additional file 1: Fig. S1.** The levels of ACE2, full-length S protein, S2 subunit, and the ratio of S2/S in transfected HEK293T cells treated with vehicle (Control), TAT or TAT-fused peptides. **Fig. S2.** Representative fluorescent microscopic image showing the presence of TAT-ACE2-2 peptide in mouse lung slices 3 days after intranasal administration. **Fig. S3.** Pseudovirus colocalizes with ACE2 in olfactory bulb. **Fig. S4.** Pseudovirus colocalizes with calbindin (CB) in olfactory bulb. **Fig. S5.** Pseudovirus does not colocalize with tyrosine hydroxylase (TH) in olfactory bulb. **Fig. S6.** Representative fluorescent microscopic image showing the presence of TAT-ACE2-2 peptide in mouse olfactory bulb 3 days after intranasal administration.

## Data Availability

The data that support the findings in this study are available from the corresponding author upon reasonable request.

## Abbreviations

SARS-CoV-2: Severe acute respiratory syndrome coronavirus 2
COVID-19: Coronavirus Disease 2019
ACE2: Angiotensin-converting enzyme 2
TMPRSS2: Transmembrane protease serine 2
TAT: Trans-Activator of Transcription
VSV: Vesicular Stomatitis Virus
GFP: Greeen Fluorescent Protein
CB: Calbindin
TH: Tyrosine Hydroxylase
MERS: Middle Ease Resiratory Syndrome
RBD: Receptor-binding domain
TMB: 3,3′,5,5′-Tetramethylbenzidine
