# Supplementary figures and images for: Development of a novel peptide to prevent entry of SARS-CoV-2 into lung and olfactory bulb cells of hACE2 expressing mice
Source: Mol Brain. 2022 Aug 9;15:71. doi: 10.1186/s13041-022-00956-1 (PMC9361269; doi:10.1186/s13041-022-00956-1)

A

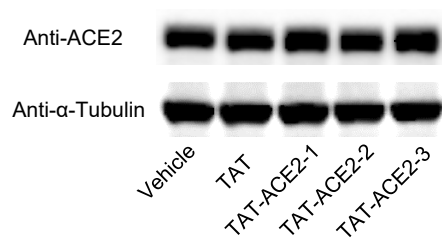

B

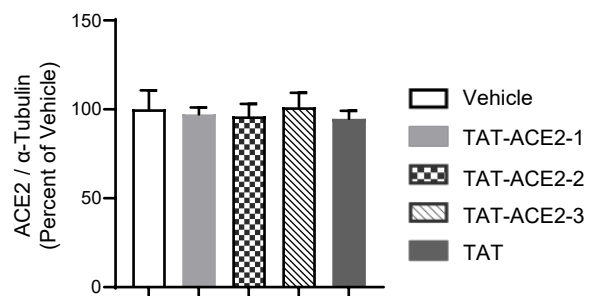

C

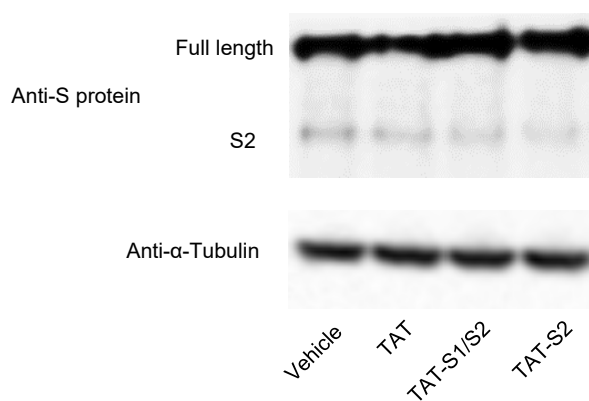

D

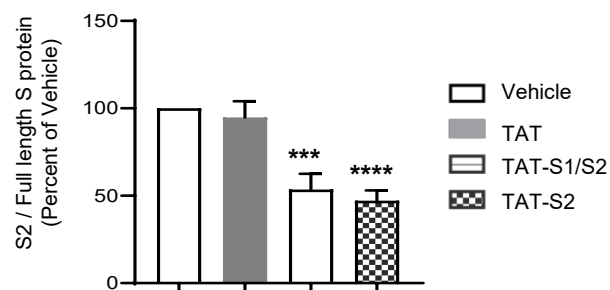

E

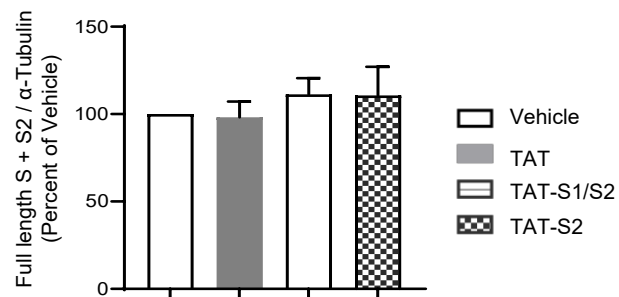

Fig. S1

## Lung

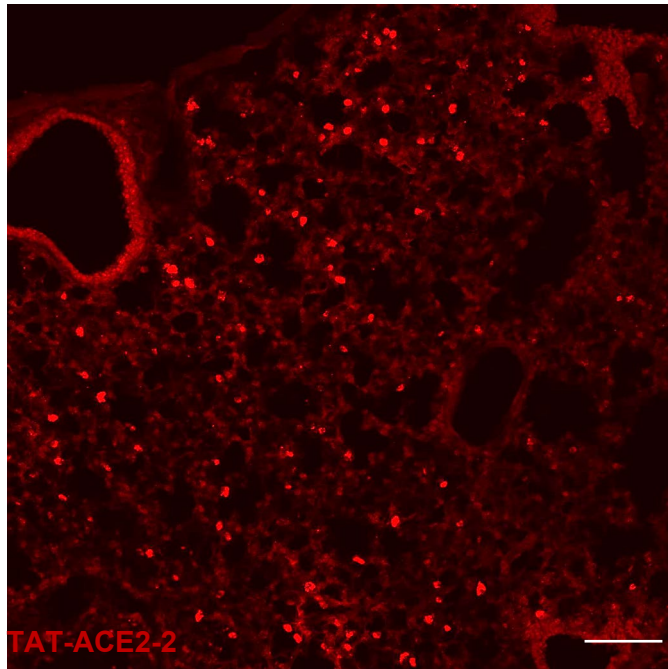

Fig. S2

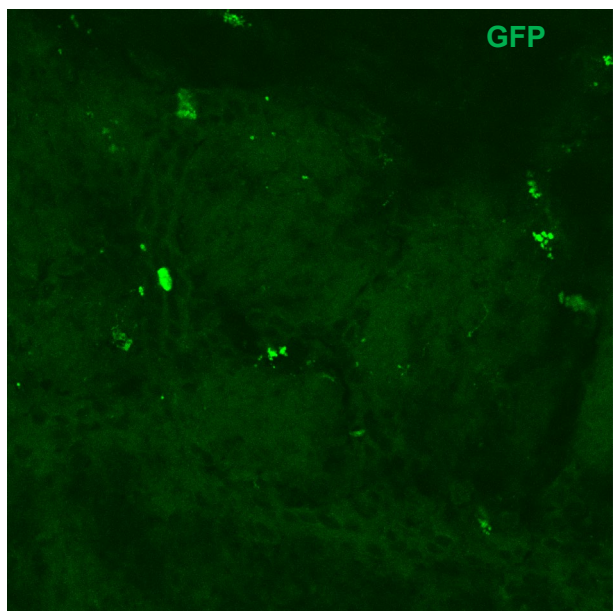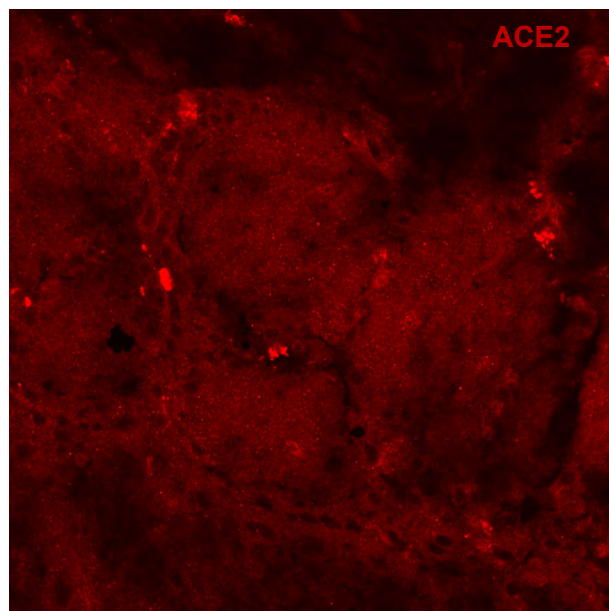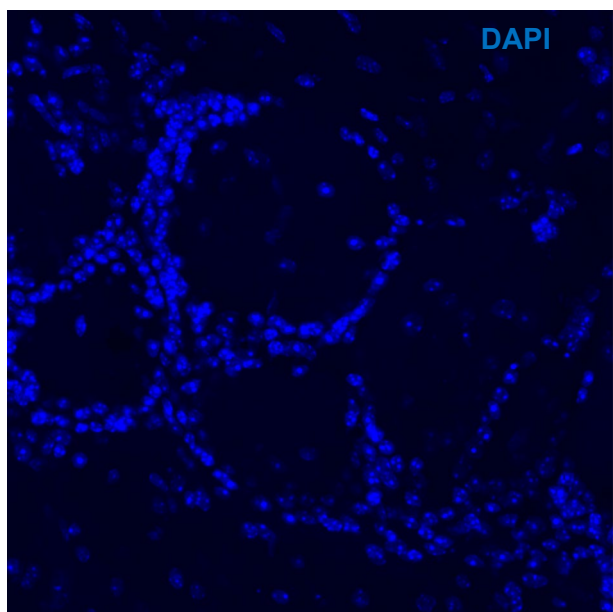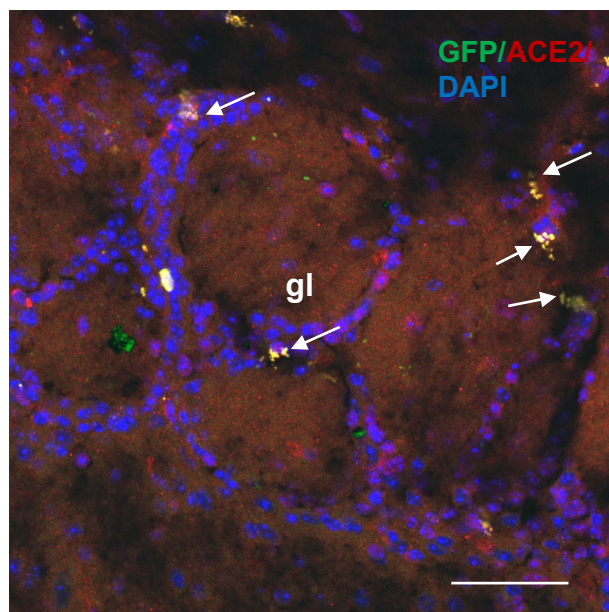

Fig. S3

A

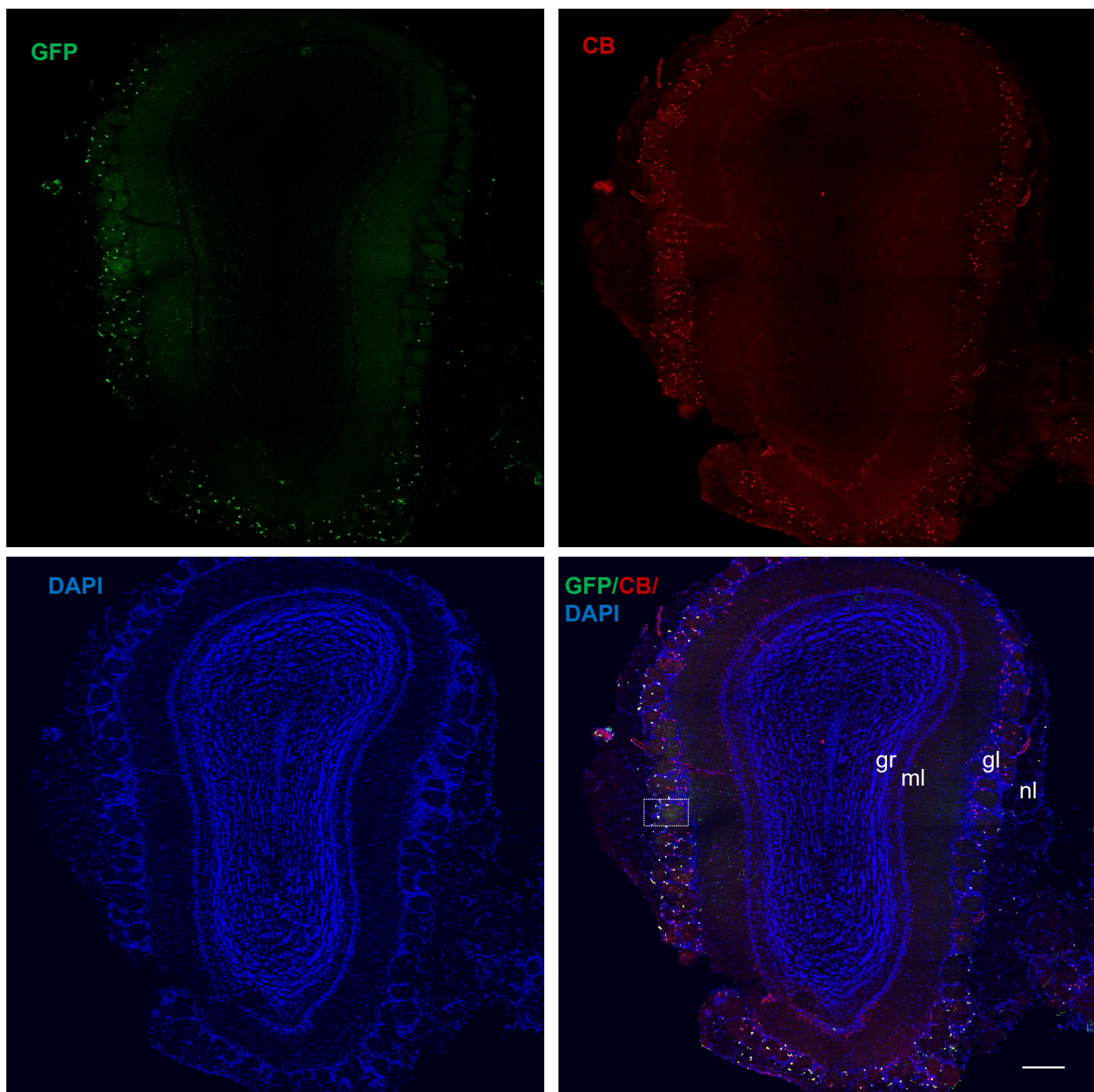

Fig. S4-1

B

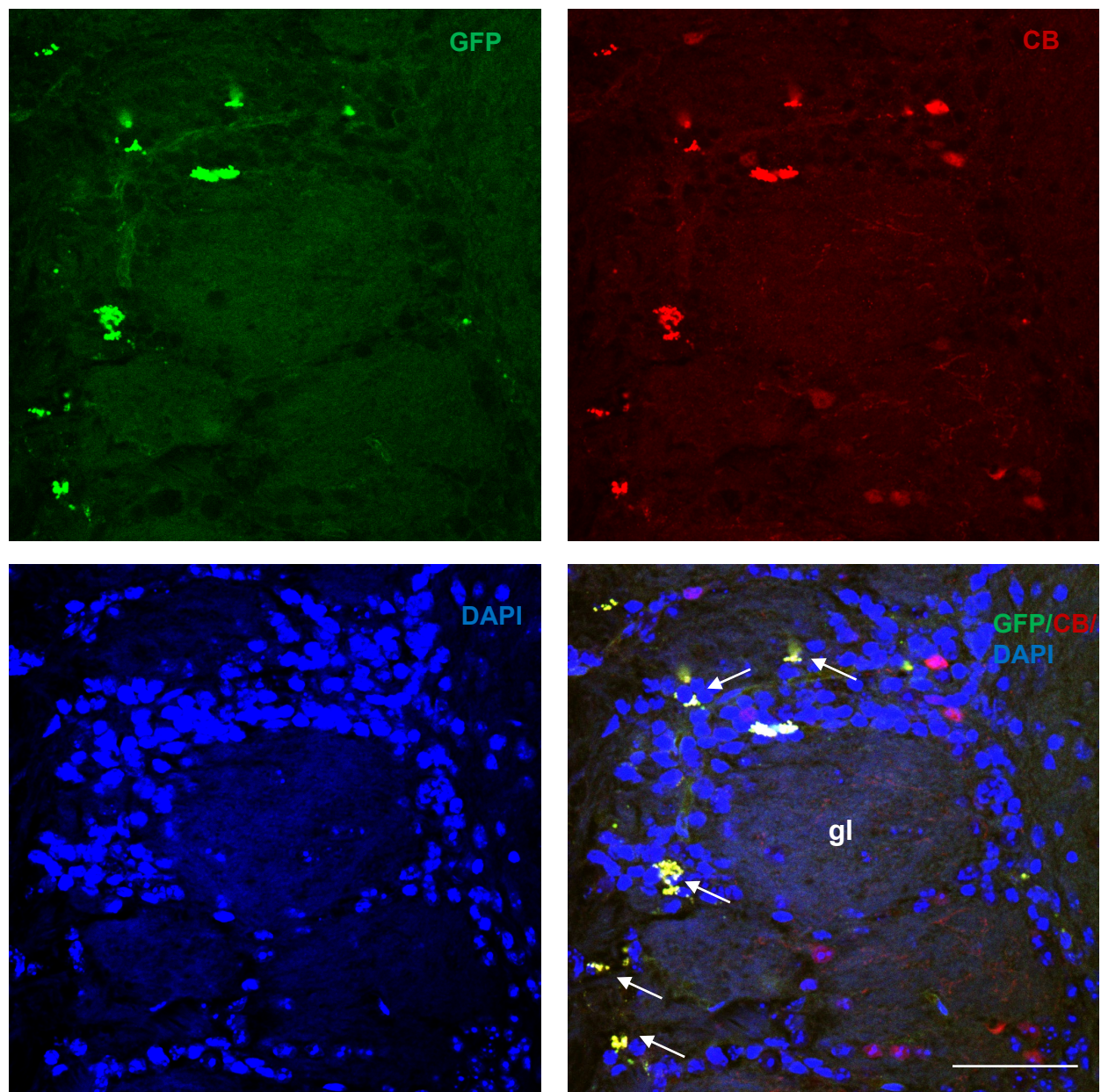

Fig. S4-2

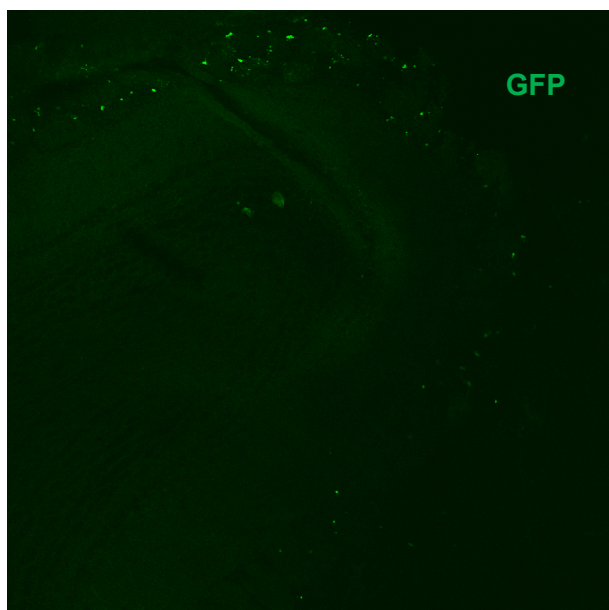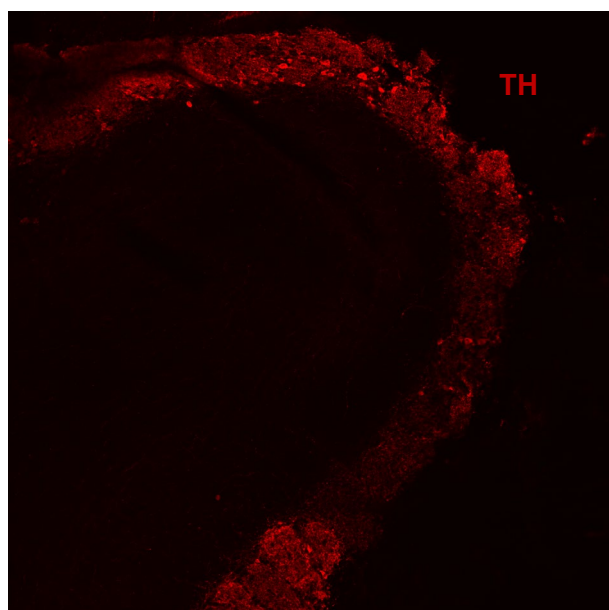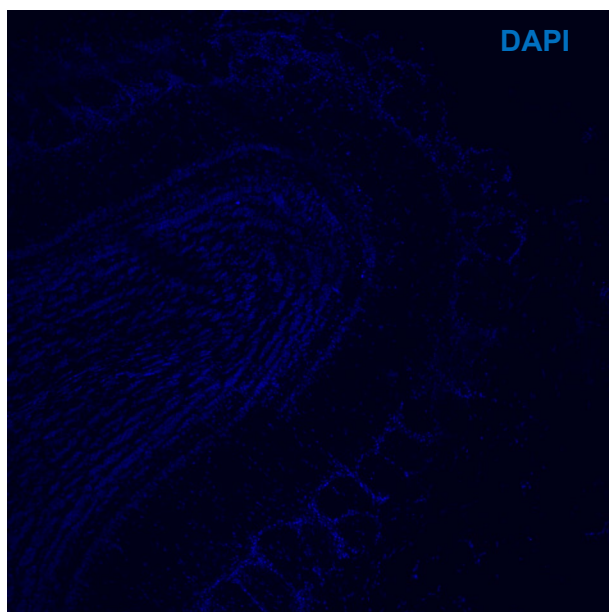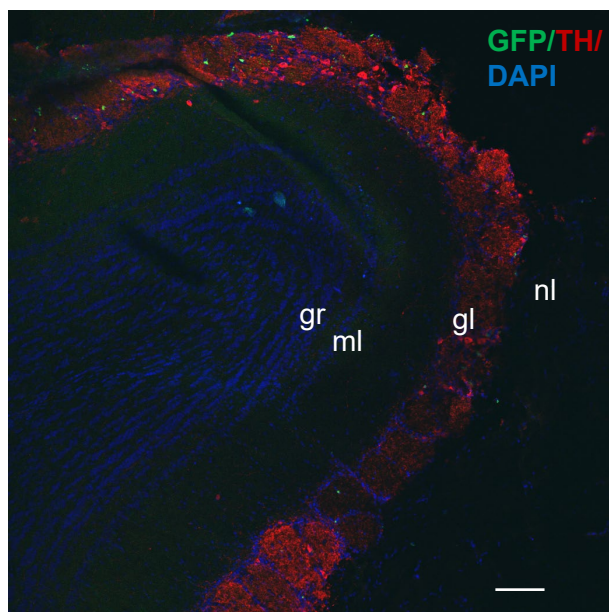

Fig. S5

OB

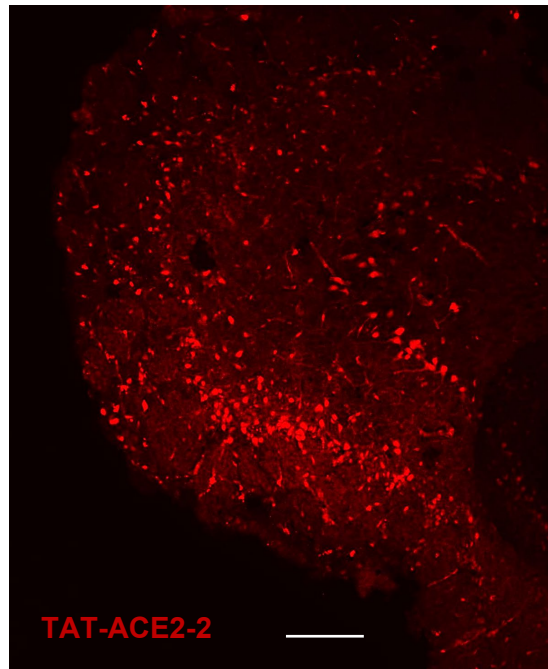

Fig. S6

Supplement: Supplementary file 1 — Additional file 1: Fig. S1. The levels of ACE2, full-length S protein, S2 subunit, and the ratio of S2/S in transfected HEK293T cells treated with vehicle (Control), TAT or TAT-fused peptides. Fig. S2. Representative fluorescent microscopic image showing the presence of TAT-ACE2-2 peptide in mouse lung slices 3 days after intranasal administration. Fig. S3. Pseudovirus colocalizes with ACE2 in olfactory bulb. Fig. S4. Pseudovirus colocalizes with calbindin (CB) in olfactory bulb. Fig. S5. Pseudovirus does not colocalize with tyrosine hydroxylase (TH) in olfactory bulb. Fig. S6. Representative fluorescent microscopic image showing the presence of TAT-ACE2-2 peptide in mouse olfactory bulb 3 days after intranasal administration. [file 13041_2022_956_MOESM1_ESM.pdf]
